# Evaluation of 980nm diode laser as adjunct to flap surgery

**DOI:** 10.6026/973206300221689

**Published:** 2026-03-31

**Authors:** Sharashchandra Patil, Kulkarni Narshima Raju, Santosh S. Kotnoor, Sapnil Gaidhankar, Soumya Reddy, Arbaz Shaikh, Kahaf Ahmadi Kadri

**Affiliations:** 1 Department of Periodontics, S.B Patil Institute for Dental Sciences and Research, Naubad, Bidar, Karnataka, India; 2 Department of Oral pathology, HKE'SN Dental College & Hospital, Kalaburagi, Karnataka, India

**Keywords:** Adjunct, chronic periodontitis, diode laser, flap surgery, open flap debridement (OFD), periodontal split-mouth study

## Abstract

The additional clinical benefit of diode laser irradiation as an adjunct to open flap debridement (OFD) in the treatment of chronic periodontitis remains uncertain. Therefore, it is of interest to evaluate the impact of diode laser irradiation during open flap debridement (OFD) for chronic periodontitis. Thirty patients with bilateral probing pocket depths of ≥5mm were randomly assigned to test and control groups. Clinical parameters such as plaque index, gingival index and probing pocket depth showed significant improvement within both groups, but no significant intergroup differences were found, except for reduced postoperative pain in the test group. The diode laser treatment was well-tolerated without complications. Thus, we show the potential benefits of diode laser irradiation as an adjunct to open flap debridement (OFD) in the treatment of chronic periodontitis.

## Background:

Periodontitis arises from a complex interplay between infectious agents, particularly bacteria and host-related factors [1]. It is widely acknowledged that periodontal disease stems from polymicrobial infections, often involving specific anaerobic microorganisms residing in the subgingival region; ultimately resulting in destruction of tooth's supporting structures [2]. Various methods are employed in periodontal treatment, including nonsurgical approaches, subgingival curettage, scaling and root planing and partial or full-thickness flap procedures, with or without osseous recontouring [3]. Traditional treatment modalities focus on mechanically eliminating subgingival plaque, disrupting biofilm and using antimicrobial agents as adjuncts. Mechanical surgical debridement targets the pockets and root surfaces compromised by periodontal damage. Marsh proposed an alternative strategy aimed at modifying the pocket environment to inhibit the proliferation of pathogenic organisms, known as the ecological plaque hypothesis [4]. Sconic and ultrasonic instruments don't directly kill periodontal pathogens but facilitate mechanical plaque and calculus removal, thereby reducing bacterial presence. Laser, an acronym for "light amplification by stimulated emission of radiation," is differentiated from ordinary light sources by its coherence, permitting it to focus precisely [5]. Diode lasers emit wavelengths ranging from 810nm to 910-980nm, which don't interact with hard dental tissues. Thus, they are ideal for soft tissue surgical applications, such as gingival cutting, coagulation, oral mucosa procedures, soft tissue curettage, or sulcular debridement [6, 7]. Therefore, it is of interest to report to assess the additional benefits of using a diode laser during OFD compared to conventional mechanical debridement, based on clinical evaluation.

## Methodology:

Patients of Stage I - III and Grade A periodontitis based on 2017 classification, belonging to both sexes who visited to the department of periodontics and oral implantology in S.B. Patil institute for dental sciences and research. The subjects were selected based on the inclusion criteria for the study. The patients were informed about the study design and the treatment procedure. Verbal and written informed consent was obtained from the patients that were going to take part in the study. Thirty systemically healthy individuals, aged between 25 and 60 years, both male and female, diagnosed with Stage I-III and Grade A periodontitis as per 2017 classification, were enrolled in study. Each participant exhibited at least 2 sites in each quadrant with PPD≥5mm, located bilaterally in either maxillary or mandibular arch, following Phase I therapy. Selection was carried out based on predefined inclusion as well as exclusion criteria. Written informed consent was taken from all participants before commencement of study. Sample size was finalized in discussion with a biostatistician, who was briefed on parameters and grouping involved in research. Study protocol was reviewed and approved by Institute Ethics Committee of S.B. Patil Institute for Dental Sciences and Research, Bidar, before the project was initiated.

Examination and collection of samples at baseline, 30 subjects having Stage I- III and Grade A periodontitis were randomized and allocated into two groups by the toss of a coin as follows:

[1] Group I - Control group receives treatment with conventional flap surgery

[2] Group II - Test group receives treatment with 980nm and 2.0-watt diode laser as an adjunct to periodontal flap surgery.

The inclusion criteria for this study required patients who were willing to participate and give consent. The patients must have been diagnosed with Stage I-III and Grade A periodontitis. Additionally, they needed to have at least 20 remaining teeth, excluding third molars. The study focused on patients who had at least two bilateral quadrants (split design) in either the maxilla or mandible, with two sites in each quadrant exhibiting probing depths of ≥ 5mm post-phase I therapy. The patients also needed to be free from any systemic illnesses and fall within the age range of 25 to 60 years. Exclusion criteria involved patients who were unable to provide information or cooperate with the dental examination. Patients with a history of underlying systemic illness, aggressive periodontitis, or who were pregnant or lactating were excluded. Additionally, patients with acute oral lesions, a history of surgical therapy within the last 6 months or those on steroid therapy and oral contraceptives were not included in the study. The clinical parameters assessed during the study included the plaque index (Silness J and Leo H, 1964), gingival index (Loe H and Silness J, 1963), Relative Clinical Attachment Level (rCAL), Probing Pocket Depth (PPD), Simplified Healing Index (SHI) and the Visual Analogue Scale (VAS). For stent fabrication, sterile, perforated metal impression trays were chosen for each patient and impressions were taken using irreversible hydrocolloid (alginate). The final casts were created by pouring dental stone. To standardize the direction of probe insertion for recording clinical parameters, an acrylic stent was fabricated in the target region using clear auto polymerizing resin, marked with red-colored paint. Probing pocket depth (PPD) was measured from the crest of marginal gingiva to the base of the periodontal pocket using a UNC15 probe. Customized occlusal stents were marked with vertical grooves to maintain consistent probe insertion in the same vertical plane. Measurements were taken with a UNC15 periodontal probe, applying a probing force of 0.75N, inserted parallel to the long axis of the tooth. The probe was "walked" around the tooth's circumference to assess pocket depth on four tooth surfaces. The site on the tooth showing the deepest probing pocket depth fulfilling the inclusion criteria was selected for the study. The procedure followed all ethical guidelines as mentioned in the 1964 Declaration of Helsinki, as revised in 2013. Written informed consent was obtained from each participant. All patients were provided with oral hygiene instructions and a standardized oral hygiene protocol was followed. Patients were instructed to brush using the roll technique with a medium-bristled toothbrush and Colgate Total toothpaste. They practiced this method under supervision until the operator was satisfied. Brushing was recommended once daily throughout the study. Manual scaling and root planing were performed. Once oral hygiene was assessed to be satisfactory, patients underwent periodontal flap procedures, with or without adjunctive diode laser treatment, 2 weeks following initial preparation. For the surgical procedures, routine blood investigations and a standardized protocol for patient safety and sterilization were followed. Necessary physician referrals were collected and noted. All procedures were performed by a single operator. Custom-fabricated acrylic stents were created on study casts and an indelible marker was used to indicate both direction and position to ensure consistent probe angulations. Flap surgery was conducted under local anesthesia with 2% Lignocaine and 1:80,000 Adrenalines. The control quadrant surgery was performed with the Open Flap Debridement (OFD) procedure. After two weeks, test side quadrant flap surgery was performed using a 980nm diode laser with a 2.0-watt, 400-micron diameter fiber-optic tip, at a frequency of 50Hz in contact mode. The laser fiber tip was moved in horizontal overlapping strokes in the sulcus and periodontal pocket for about 20 seconds and 12 strokes in two phases and debridement with curettes and root planing were performed using ultrasonic devices. Postoperative care involved standard instructions, with prescriptions for antibiotics and anti-inflammatory medications. Amoxicillin 500mg was prescribed three times daily, Diclofenac sodium 100mg twice daily and Metronidazole 400mg three times daily for 5 days. Pantoprazole and Domperidone were prescribed to prevent gastric discomfort. A 0.2% Chlorhexidine Digluconate mouth rinse was recommended twice daily for one week. Patients were advised to refrain from brushing the treated area for one week. During follow-up visits, they were asked about postoperative pain or bleeding and oral hygiene instructions were reinforced. Postsurgical assessments were conducted at baseline and on the 14th day, which included VAS and SHI. Clinical parameters such as rCAL, PPD, PI and GI were assessed at baseline 3 months and 6 months postoperatively.

## Results:

Study sample comprised 30 patients, including 28 women and 2 men, aged between 25 and 60 years. A total of 60 sites were treated-30 in test group and 30 in control group-using a split-mouth design. For evaluating clinical parameters, average of two sites in each quadrant (either test or control) per patient was used. Mean values along with standard deviations (SD) were recorded at baseline, on 7th and 14th days and at 3 and 6 months. Results were presented as mean ± standard deviation (SD). Intragroup comparisons (from baseline to subsequent intervals) were carried out by employing ANOVA and paired t-tests, while intergroup comparisons (test vs. control) were assessed with unpaired t-tests. A p value<0.05 was considered statistically significant. In control group, intragroup comparison of PI showed mean scores of 1.87±0.37 at baseline, 1.26±0.14 at 3 months and 0.94±0.45 at 6 months (Table 1 - see PDF). On intragroup comparison, PI in test group at baseline, 3months & 6 months, mean PI scores were 1.79±0.34, 1.12±0.12 & 0.93±0.14, respectively (Table 2 - see PDF). On comparison, PI shows a proportional decrease from baseline to 3 months & 6 months, but P value of 0.00 indicates that these changes are statistically significant. On intergroup comparison at baseline, both test & control have similar mean PI values of 1.79±0.34 and 1.87±0.37 with a high p-value of 0.41, indicating no significant difference between groups at baseline. 3 months control and test had mean PI values of 1.26±0.14 and 1.12±0.12, which is again similar with p-value of 0.514, showing insignificant difference. At 6 months, test group shows a higher mean PI value 0.93±0.14 compared with control group 0.94±0.12, but p-value is 0.641, indicating insignificant difference at 5% significance level (Table 3 - see PDF). On intragroup comparison in control group, GI at baseline, 3 months & 6 months mean GI scores were 1.47±0.27, 1.16±0.26 and 0.94±0.10, respectively (Table 4 - see PDF). On comparison, GI significantly decreases from baseline to 3 months & 6 months, with P-value of 0.000, suggesting statistically significant improvement in gingival health over time. On intragroup comparison of GI in test group at baseline, 3months and 6 months, mean GI scores were 1.44±0.29, 1.13±0.13 and 0.91±0.13, respectively (Table 5 - see PDF). On comparison, GI shows a significant reduction from baseline to 6 months, with P-value of 0.000, showing significant improvement of gingival health over time. On intergroup comparison at baseline, both control and test groups have nearly identical mean GI values of 1.47±0.27 and 1.44±0.29, with p-value of 0.666, suggesting insignificant difference. At 3 months, mean GI values of control & test group 1.16±0.26 and 1.13±0.13 remain similar with p-value of 0.580, demonstrating insignificant difference. At 6 months, mean GI values of control and test group were found to be 0.94±0.10 & 0.91±0.13, which are close to p-value of 0.237, suggesting insignificant difference (Table 6 - see PDF). On intragroup comparison in control group of Visual Analogue Scale at baseline, 7th day & 14th day, mean VAS scores were 3.00±0.87, 2.23±1.35 & 0.80±0.76, respectively (Table 7 - see PDF). VAS scores show a significant reduction in pain perception from baseline to 7 days & 14 days, with P-value of 0.000, suggesting significant decrease in pain over time. On intragroup comparison of test group visual analogue scale at baseline, 7th days & 14th days, mean VAS scores were 3.20±0.92, 1.60±0.49 & 0.63±0.55, respectively (Table 8 - see PDF). On intergroup comparison at baseline, test group has a slightly lower mean VAS score of 3.20±0.92 compared with control group 3.00±0.87, but p-value is 0.392, suggesting insignificant difference. On 7th day, mean VAS scores of control &test (2.23±1.35 & 1.60±0.49 are almost same, with p-value of 0.020, demonstrating significant difference. At 14th day, control group has a lower mean VAS score of 0.80±0.76, compared with test group 0.63±0.55, with p-value of 0.337, indicating no significant difference (Table 9 - see PDF).

Table 10 (see PDF) shows a statistically significant reduction in the mean Simplified Healing Index (SHI) score in the control group from 7 days (1.8 ± 0.40684) to 14 days (1.1 ± 0.30513), as determined by the paired t-test (p < 0.005) (Table 10 - see PDF). The simplified healing index shows a significant improvement from 7-14 days, with P-value of less than 0.005, exhibiting statistically significant enhancement in healing over time. On intragroup comparison in test group of simplified healing index at 7th days & 14th days, mean SHI scores were 1.78±0.48 & 1.03±0.17, respectively (Table 11 - see PDF). The simplified healing index shows a significant improvement from 7-14 days, with P-value of less than 0.005, demonstrating statistically significant enhancement in healing over time. On intergroup comparison at 7th day, both groups have similar scores of 1.80±0.48 & 1.80±0.40 with p-value of 1.00, suggesting insignificant difference. At 14 days, test group has a mean score 1.03±0.18 compared to control group 1.10±0.30 with p-value of 0.309, suggesting insignificant difference between groups (Table 12 - see PDF). On intragroup in control group comparison of PPD at baseline, 3 months & 6 months, mean PPD scores were 7.31±0.97, 3.56±0.46 & 3.25±0.45, respectively (Table 13 - see PDF). PPD significantly decreases from baseline to 3 months & 6 months, with P-value of 0.000, indicating significant improvement in periodontal pocket depth. On intragroup in test group comparison of PPD at baseline, 3months & 6 months, mean PPD scores were 7.5333±1.08, 3.65±0.397 & 3.23±0.28, respectively (Table 14 - see PDF). PPD significantly decreases from baseline to 3 month & 6 months, with P-value of 0.000, indicating substantial reduction in periodontal pocket depth. On intergroup comparison at baseline, both control & test groups have almost identical mean PPD values of 7.31±0.97 & 7.53±1.08, with p-value of 0.419, suggesting insignificant difference. At 3 months, mean PPD values are similar, 3.56±0.46 in control group & 3.65±0.39 in test group, with p-value of 0.460, showing insignificant difference. At 6 months, both groups have almost same mean PPD value 3.25±0.45 & 3.23±0.28 with p-value of 0.865, suggesting insignificant difference (Table 15 - see PDF). On intragroup in control group comparison of relative clinical attachment level at baseline, 3 months & 6 months, mean rCAL scores were 8.76±1.22, 7.78±1.19 & 7.56±1.22, respectively (Table 16 - see PDF). The relative clinical attachment level shows a significant improvement from baseline to 3 months & 6 months, with P-value of 0.001, indicating significant enhancement in clinical attachment over time. On intragroup in test group comparison of rCAL at baseline, 3 months & 6 months, mean rCAL scores were 9.18±0.98, 8.11±1.09 & 8.06±0.99 respectively (Table 17 - see PDF). rCAL level shows a significant improvement from baseline to 3months & 6 months, with P-value of 0.000, indicating significant enhancement of clinical attachment over time. On intergroup comparison at baseline control & test groups have similar mean rCAL values of 8.76±1.22 & 9.18±0.98 with p-value of 0.152, suggesting insignificant difference. At 3 months control & test group have mean rCAL values of 7.78±1.19 & 8.11±1.09, with p-value of 0.265, demonstrating insignificant difference. At 6 months mean rCAL values of control & test group are 7.56±1.22 & 8.06±0.99, with p-value of 0.89, indicating insignificant difference (Table 18 - see PDF).

## Discussion:

Chronic Periodontitis (CP) is a multifactorial disease characterized by an inflammatory response to periopathogens. Treatment options include both surgical and non-surgical methods, each with distinct advantages and limitations. Non-surgical treatment, primarily scaling and root planing (SRP), remains the most effective for periodontal pockets deeper than 4mm. However, SRP's long-term success is inconsistent due to factors like stress, smoking and systemic illnesses, leading to the exploration of alternative therapies, including laser treatments. Laser therapy, particularly using diode lasers, has shown promising results, offering additional benefits such as bacterial reduction and tissue healing [8]. In a study by Karthikeyan *et al.* (2019) [9], diode lasers were found to have no significant impact on plaque index (PI) values, which aligns with findings of our study suggesting that laser therapy does not affect plaque accumulation but can effectively control gingival inflammation. Another study by Meyer *et al.* (2017) [10] corroborated this by demonstrating that gingivitis can be significantly reduced with strict oral hygiene and periodontal treatment. Additionally, laser treatments were linked to significant reductions in gingival inflammation (GI) and were associated with reduced postoperative pain, as reflected by lower Visual Analogue Scale (VAS) scores in the laser-treated group. Clinical parameters such as Probing Pocket Depth (PPD) and Clinical Attachment Level (CAL) were improved in both the laser and control groups, aligning with results from several studies, including which showed that periodontal therapy can enhance clinical outcomes [11]. Overall, the findings suggest that diode laser therapy, as an adjunct to conventional periodontal therapy, can effectively reduce inflammation, enhance healing and provide additional clinical benefits in managing CP.

## Conclusion:

Diode laser therapy combined with Open Flap Debridement shows promise in improving clinical attachment levels and reducing probing pocket depth. The results support its incorporation into clinical practice for periodontal conditions. Further research with larger sample sizes is needed to better understand its clinical effectiveness.
